# The iRhom2/ADAM17 Axis Attenuates Bacterial Uptake by Phagocytes in a Cell Autonomous Manner

**DOI:** 10.3390/ijms21175978

**Published:** 2020-08-19

**Authors:** Anke Seifert, Justyna Wozniak, Stefan Düsterhöft, Petr Kasparek, Radislav Sedlacek, Stephan Dreschers, Thorsten W. Orlikowsky, Daniela Yildiz, Andreas Ludwig

**Affiliations:** 1 Institute of Molecular Pharmacology, Medical Faculty, RWTH Aachen University, 52074 Aachen, Germany; aseifert@ukaachen.de (A.S.); jwozniak@ukaachen.de (J.W.); sdusterhoft@ukaachen.de (S.D.); 2 Czech Centre for Phenogenomics, Institute of Molecular Genetics of the Czech Academy of Sciences, 25250 Vestec, Czech Republic; petr.kasparek@img.cas.cz (P.K.); radislav.sedlacek@img.cas.cz (R.S.); 3 Department of Neonatology, University Children’s Hospital, 52074 Aachen, Germany; sdreschers@ukaachen.de (S.D.); torlikosky@ukaachen.de (T.W.O.); 4 Institute of Experimental and Clinical Pharmacology and Toxicology, PZMS, ZHMB, Saarland University, 66424 Homburg, Germany

**Keywords:** metalloproteinase, ADAM17, iRhom2, bacterial phagocytosis, inflammation, infection, shedding, chemokines, phagocytes

## Abstract

Uptake of bacteria by phagocytes is a crucial step in innate immune defence. Members of the disintegrin and metalloproteinase (ADAM) family critically control the immune response by limited proteolysis of surface expressed mediator molecules. Here, we investigated the significance of ADAM17 and its regulatory adapter molecule iRhom2 for bacterial uptake by phagocytes. Inhibition of metalloproteinase activity led to increased phagocytosis of pHrodo labelled Gram-negative and -positive bacteria (*E. coli* and *S. aureus*, respectively) by human and murine monocytic cell lines or primary phagocytes. Bone marrow-derived macrophages showed enhanced uptake of heat-inactivated and living *E. coli* when they lacked either ADAM17 or iRhom2 but not upon ADAM10-deficiency. In monocytic THP-1 cells, corresponding short hairpin RNA (shRNA)-mediated knockdown confirmed that ADAM17, but not ADAM10, promoted phagocytosis of *E. coli*. The augmented bacterial uptake occurred in a cell autonomous manner and was accompanied by increased release of the chemokine CXCL8, less TNFα release and only minimal changes in the surface expression of the receptors TNFR1, TLR6 and CD36. Inhibition experiments indicated that the enhanced bacterial phagocytosis after ADAM17 knockdown was partially dependent on TNFα-activity but not on CXCL8. This novel role of ADAM17 in bacterial uptake needs to be considered in the development of ADAM17 inhibitors as therapeutics.

## 1. Introduction

Proteases of the a disintegrin and metalloproteinase (ADAM)-family play a key role in many physiological and pathological processes during development, inflammation and cancer [1,2]. The most prominent members of this family, ADAM10 and ADAM17 are expressed as type-1 transmembrane molecules consisting of an N-terminal prodomain, a metalloproteinase domain, a disintegrin-like domain, a membrane-proximal domain (MPD), a stalk region, a transmembrane helix and a cytoplasmic C-terminal region [3]. Many functions of cell surface-expressed ADAMs can be attributed to their ability to mediate proteolytic shedding of several transmembrane proteins via their metalloproteinase domain. The fact that systemic absence of ADAM10 or ADAM17 in mice causes death early in development or shortly after birth shows the importance of these proteases and necessitates studying conditional or cell specific knockouts [4,5].

ADAM10 and ADAM17 are regulated at multiple levels including their maturation, trafficking and localisation. Pseudoproteases of the rhomboid superfamily iRhom1 (RHBDF1) and iRhom2 (RHBDF2) were identified as key interactors for ADAM17 that mediate trafficking of the ADAM17 proform from the ER to the Golgi [6,7,8]. Subsequently, ADAM17 undergoes maturation by furin-like proteases in the Golgi and can be transported to the cell surface, where the shedding process would occur. In immune cells, this trafficking of ADAM17 mainly depends on iRhom2, since these cells only express iRhom2 but not iRhom1, which is present in most other cells [6,9,10]. Mutations leading to inactivation or hyperactivation of ADAM17 or iRhom2 in humans are extremely rare. Those patients can survive but suffer from recurrent infections or tylosis with oesophageal cancer, highlighting the importance of the iRhom2/ADAM17 axis in immunity, host defence and cancer [11,12,13].

Due to the fact that ADAM10 and ADAM17 shed a number of different surface molecules, it is very likely that the effects in vivo result from either activation or abrogation of many different signalling pathways elicited by these substrates. For example, ADAM17 can promote the inflammatory response by shedding of TNFα. This pathway is, for example, critically involved in lipopolysaccharide (LPS)-induced septic shock [14]. Other proinflammatory pathways include the shedding of IL-6R, adhesion molecules like E-cadherin and JAM-A and the transmembrane chemokines CXCL16 and CX3CL1 by ADAM17 and ADAM10 [15,16,17,18]. Via shedding of EGFR ligands, ADAM17 also contributes to toll-like receptor (TLR)-mediated transactivation of EGFR inducing enhanced inflammatory mediator production (e.g., CXCL8) [19]. However, ADAM17 can also suppress the inflammatory cytokine signalling by shedding of TNFR1/2, or suppress leukocyte extravasation by cleavage of L-selectin [20,21]. Additionally, ADAM10 and ADAM17 are involved in the cleavage of pattern recognition receptors (PRR) like the scavenger receptors CD163, CD36 and CXCL16 [22,23,24,25] and the toll-like receptor TLR2 [26]. Moreover, it was shown that increased release of TNFR due to ADAM17 activation by mannose-capped lipoarabinomannan facilitates the maintenance of Mycobacterium tuberculosis infection [27], and that myeloid ADAM17-deficiency leads to a reduced bacterial burden in the inflamed lung upon sepsis caused by Gram-negative bacteria [28,29]. Therefore, ADAM17 and ADAM10 represent crucial hubs for many signalling pathways that either promote or limit inflammatory signalling and eventually contribute to the final immune response in a complex manner. Moreover, for each cellular function during the immune response, the importance of the different proteases can vary considerably. A recent study showed that ADAM17-deficiency in microglia leads to higher uptake of apoptotic cells [30], but implications for antibacterial defense were not investigated To our knowledge, a direct role of the proteases in bacterial uptake via isolated leukocytes has not yet been described.

In the present study, we investigated the significance of ADAM10 and ADAM17 for bacterial uptake by human and murine phagocytes. Inhibition or short hairpin RNA (shRNA)-mediated downregulation of ADAM17 leads to increased phagocytosis of pHrodo labelled Gram-negative and -positive bacteria by monocytic cell lines or primary phagocytes. Moreover, deficiency or inactivation of the iRhom2/ADAM17 axis in bone marrow-derived macrophages (BMDMs) enhances the uptake of heat-inactivated and living *Escherichia coli* (*E. coli*). In THP-1 cells the augmented bacterial uptake preferentially occurs in cells with ADAM17 knockdown and is accompanied by an increased release of CXCL8. The enhanced bacterial phagocytosis after knockdown of ADAM17 can be reduced by infliximab and etanercept indicating that this is partially dependent on TNFα-signalling.

## 2. Results

### 2.1. Inhibition of ADAM Proteases Increases Uptake of pHrodo Labelled Bacteria by Human and Murine Phagocytes

**Figure 1:**
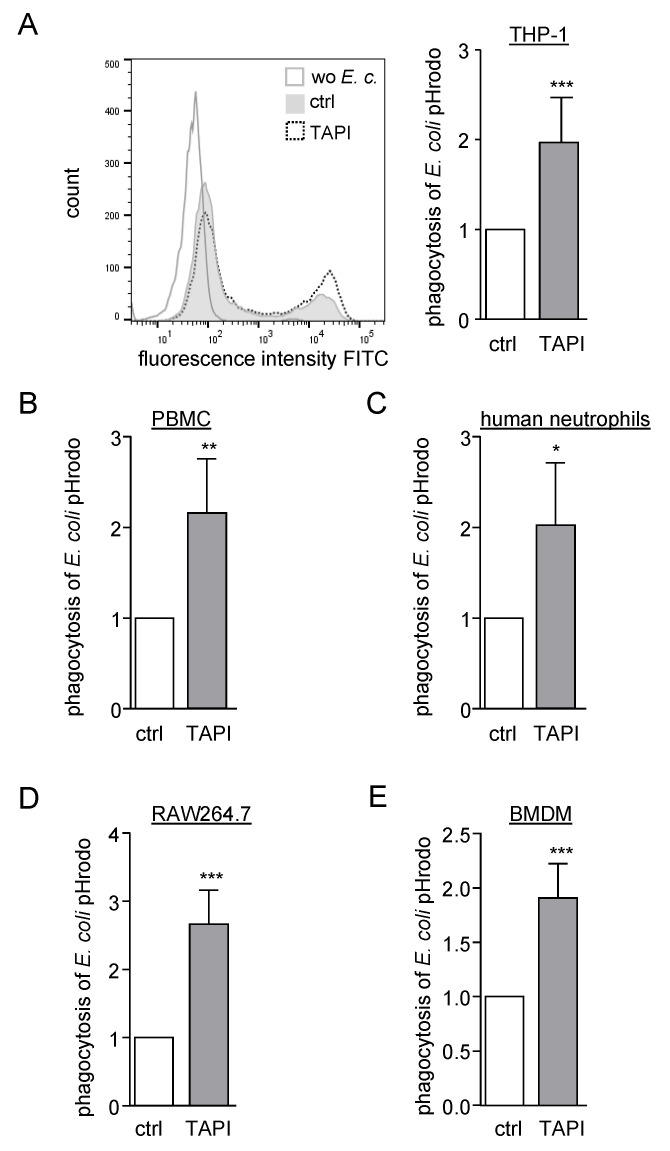
The a disintegrin and metalloproteinase (ADAM) inhibitor TAPI-1 increases uptake of *E. coli* pHrodo, (**A**–**E**) THP-1 cells (**A**), human peripheral blood mononuclear cells (PBMCs) (**B**), human neutrophils (**C**), murine RAW264.7 cells (**D**) and murine bone marrow-derived macrophages (BMDMs) (**E**) were preincubated with 10 µM TAPI or 0.1% DMSO as vehicle control (ctrl), exposed to *E. coli* pHrodo green and assayed for phagocytosis by flow cytometry. Representative histogram overlay of the fluorescence signal from *E. coli* pHrodo treated and untreated (wo *E. c*.) THP-1 cells is shown in (**A**). The geometric mean fluorescence for each cell type was calculated in relation to that of the respective control and summarized as mean and SD of at least four independent experiments (**A**) *n* = 8, (**B**) *n* = 4, (**C**) *n* = 5, (**D**) *n* = 8, (**E**) *n* = 7. Significant differences compared to the respective control are indicated as asterisks (* *p* < 0.05, ** *p* < 0.01, *** *p* < 0.001).

To analyse the influence of ADAM proteases on particle uptake different human phagocytic cell lines and primary cells (THP-1, freshly isolated peripheral blood mononuclear cells (PBMCs) and neutrophils) as well as murine phagocytes (RAW264.7 and BMDMs) were preincubated with the metalloproteinase inhibitor TAPI-1 which is a well-known ADAM17 inhibitor but also blocks ADAM10. Subsequently, cells were treated with pHrodo labelled, heat-inactivated *E. coli* (Supplementary Figure S1A). Since pHrodo is non-fluorescent at neutral pH and becomes fluorescent at acidic pH, the uptake of *E. coli* pHrodo in acidic compartments of phagocytes can be measured by flow cytometry in terms of geometric mean fluorescence intensity. All used cell lines and primary cells showed an approximately two-fold increase in phagocytosis when they were treated with TAPI-1. (Figure 1A–E).

Exemplarily for THP-1 cells, we also analysed the percentage of phagocytosis positive cells. Here, the same difference as in the analysis of the geometric mean intensity was observed (Supplementary Figure S1B). Since the geometric mean quantifies the amount of particle uptake per cell and can still detect differences when nearly all cells are phagocytosis positive, this method was chosen for subsequent experiments.

In the next experiments, we used Gram-positive *S. aureus* pHrodo green particles and found that their uptake was also increased with TAPI-1 but to a lesser extent than it was seen for the phagocytosis of Gram-negative *E. coli* pHrodo green particles (Supplementary Figure S1C).

### 2.2. ADAM17 Deficiency in BMDMs Increases Phagocytosis of Bacteria Ex Vivo

**Figure 2:**
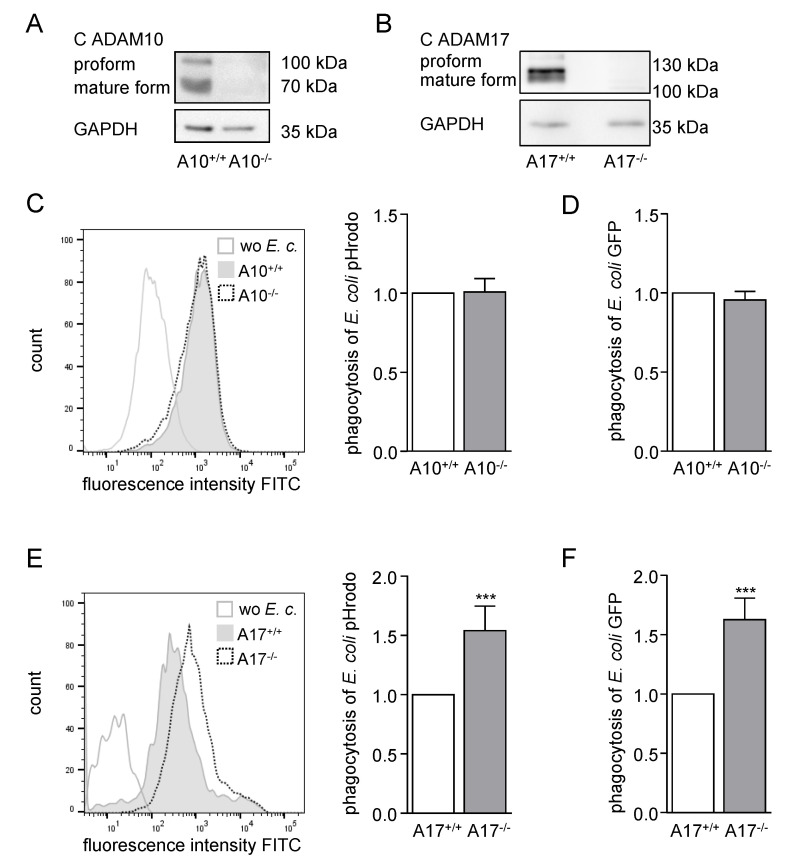
Deficiency of ADAM17 in BMDMs leads to increased phagocytosis of *E. coli.* (**A**,**B**) BMDMs from Vav-*Adam10*^−/−^ mice (A10^−/−^) (**A**) or Vav-*Adam17*^−/−^ mice (A17^−/−^) (**B**) and their respective wild type littermates (A10^+/+^, A17^+/+^) were analysed for ADAM10 (**A**) or ADAM17 (**B**) protein expression by western blot using antibodies against the C-terminus. Glyceraldehyde-3-phosphate dehydrogenase (GAPDH) was detected as loading control. (**C**–**F**) BMDMs as described in (**A**,**B**) were exposed to *E. coli* pHrodo green (**C**,**E**) or living *E. coli* expressing GFP (**D**,**F**) and assayed for phagocytosis by flow cytometry. Representative histograms of the particle uptake in ADAM10-(**C**) or ADAM17-deficient (**E**) BMDMs compared to wild type BMDMs are shown in (**C**) and (**E**). The geometric mean fluorescence of the cells was calculated in relation to that of the respective wild type litter control and summarized as mean and SD of at least four independent experiments (**C**) *n* = 4, (**D**) *n* = 5, (**E**) *n* = 8, (**F**) *n* = 5). Significant differences compared to the respective control are indicated as asterisks (*** *p* < 0.001).

To further elucidate the influence of ADAM10 and ADAM17 on bacterial phagocytosis, BMDMs were generated out of Vav-*Adam10*^−/−^mice, Vav-*Adam17*^−/−^ mice and their wild type littermates (Supplementary Figure S2A). The knockout of ADAM10 or ADAM17 in the BMDMs was confirmed at the gene and protein level (Supplementary Figure S2C,F and Figure 2A,B).

Whereas knockout of ADAM10 did not influence the uptake of heat-inactivated pHrodo labelled bacteria (Figure 2C; Supplementary Figure S2E), the deficiency of ADAM17 in BMDMs led to significantly increased phagocytosis of *E. coli* and *S. aureus* pHrodo (Figure 2E; Supplementary Figure S2H). Of note, the effect of ADAM17 knockout on the uptake of *E. coli* particles was a little higher than on the uptake of *S. aureus* pHrodo.

To confirm these findings, living GFP-expressing *E. coli* were probed for uptake by BMDMs. Again, phagocytosis was clearly enhanced when these cells lacked ADAM17 (Figure 2F; Supplementary Figure S2B,G) but not when ADAM10 was absent (Figure 2D; Supplementary Figure S2D). Taken together, we concluded that ADAM17 but not ADAM10 has a suppressive influence on bacterial phagocytosis.

### 2.3. Deficiency or Inactivation of iRhom2 Leads to Enhanced Phagocytosis Ex Vivo

**Figure 3:**
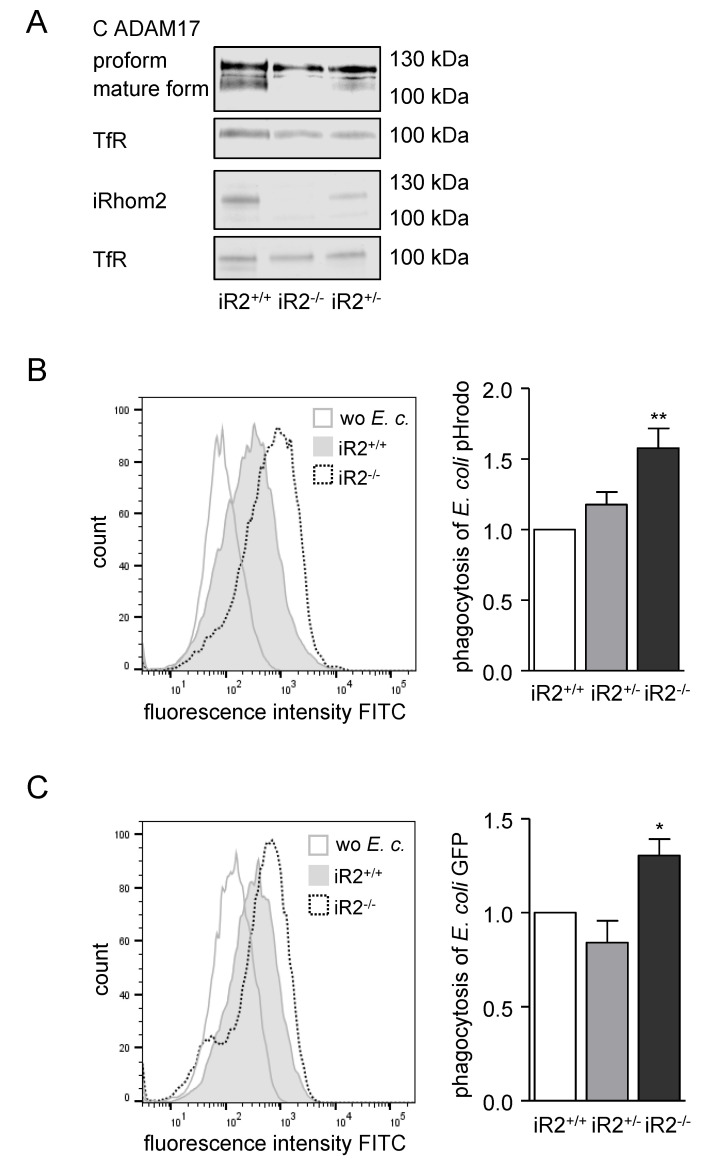
Deficiency or inactivation of iRhom2 in BMDMs enhances phagocytosis of *E. coli*, (**A**) BMDMs from heterozygous (iR2^+/−^) or homozygous (iR2^−/−^) *iRhom2* KO mice and their wild type littermates (iR2^+/+^) were analysed for their iRhom2 and ADAM17 protein expression by Western blot. Transferrin receptor was detected as loading control. (**B**,**C**) BMDMs as described in A were exposed to *E. coli* pHrodo green (**B**) or living *E. coli* expressing GFP (**C**) and assayed for phagocytosis by flow cytometry. Results are shown as representative histograms and as geometric mean fluorescence of the cells in relation to that of the littermate control. Data are shown as mean and SD of three independent experiments. Significant differences compared to the respective control are indicated as asterisks (* *p* < 0.05, ** *p* < 0.01).

It is known that iRhoms are indispensable for the maturation and activity of ADAM17 [3,6,8,9]. While iRhom1 is expressed in most cell types, iRhom2 is dominantly expressed in immune cells. For that reason, we generated BMDMs out of *iRhom2^−^*^/*−*^ and *iRhom2^+/−^* mice and their wild type littermates. Knockout of iRhom2 was verified at the protein and gene expression level (Figure 3A, Supplementary Figure S3A). Deficiency of iRhom2 led to a clear, dose-dependent downregulation of iRhom2 mRNA and protein expression. Strikingly, the mature form of ADAM17 was reduced in heterozygous—and nearly lost in homozygous BMDMs—confirming the importance of iRhom2 for the maturation of ADAM17 in BMDMs (Figure 3A). Consistent with our previous results, phagocytosis assays with homozygous iRhom2-deficient BMDMs showed that uptake of *E. coli* pHrodo and living *E. coli* was significantly increased compared to their respective wild type controls (Figure 3B,C). Moreover, phagocytosis of *E. coli* pHrodo was also increased in THP-1 iRhom2 knockdown cells (Supplementary Figure S3B,C). Worth mentioning, heterozygous iRhom2 knockout did not lead to a change in phagocytosis of the BMDMs, implying that a lesser amount of mature ADAM17 is still sufficient to keep the phagocytosis rate in a regular range. Only if almost no mature ADAM17 was present in the BMDMs, the bacterial uptake was augmented (Figure 3B,C). Still, these results support the hypothesis, that the iRhom2/ADAM17 axis attenuates bacterial phagocytosis.

### 2.4. Knockdown of ADAM17 in THP-1 Cells Enhances Phagocytosis and CXCL8 Release

**Figure 4:**
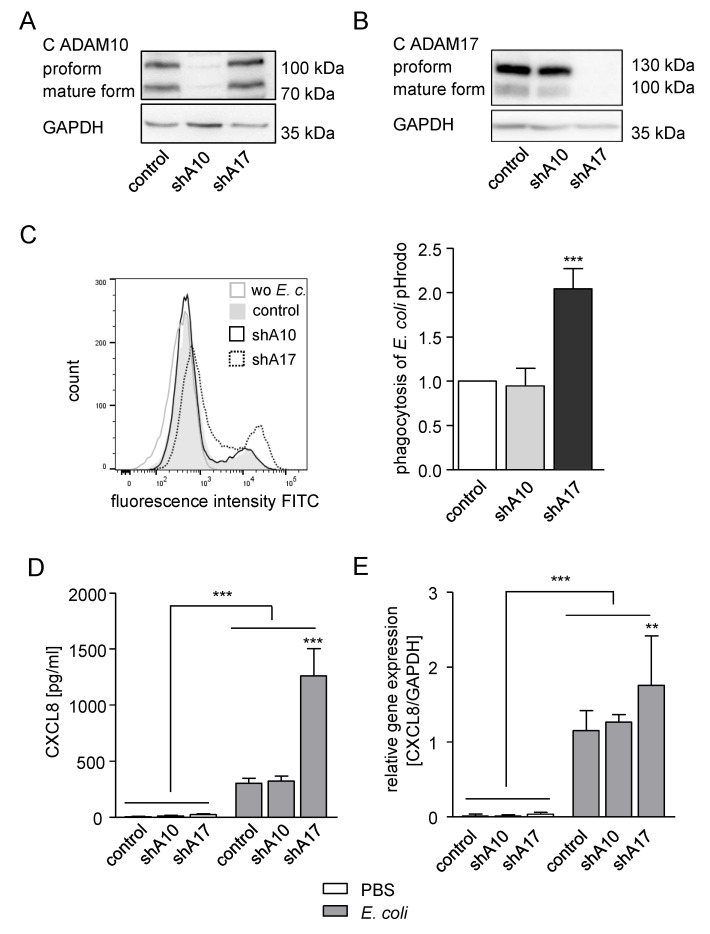
Knockdown of ADAM17 enhances phagocytosis of *E. coli* and CXCL8 release, (**A**,**B**) THP-1 cells were transduced with lentivirus encoding control–shRNA (Short hairpin RNA) (control), ADAM10–shRNA (shA10) or ADAM17–shRNA (shA17) and analysed for ADAM10 (**A**) and ADAM17 (**B**) knockdown by Western blot. GAPDH served as loading control. (**C**,**E**) THP-1 cells transduced as described in (**A**,**B**) were exposed to *E. coli* pHrodo green and assayed for phagocytosis by flow cytometry (**C**), CXCL8 release by ELISA (**D**) and CXCL8 mRNA expression by RT-qPCR (**E**). Quantitative data were calculated in relation to that of the indicated control. Data are shown as mean and SD or as one representative of at least three independent experiments (**C**) *n* = 6; (**D**,**E**) *n* = 3. Significant differences compared to the respective control are indicated as asterisks (** *p* < 0.01, *** *p* < 0.001) and additional comparisons are specified by bars.

For further detailed analysis of our observations, we decided to use THP-1 cells. To first confirm the role of ADAM17 in phagocytosis by these cells, we performed shRNA-mediated ADAM10 or ADAM17 knockdown in THP-1 cells, which was controlled at the mRNA and protein level (Supplementary Figure S4A,B; Figure 4A,B). As expected, silencing of ADAM10 expression in THP-1 cells did not have an impact on the uptake of *E. coli* pHrodo particles compared to control cells. In contrast, knockdown of ADAM17 led to clearly enhanced phagocytosis of *E. coli* pHrodo in the THP-1 cells (Figure 4C). We then questioned whether the release of CXCL8 and TNFα during or after phagocytosis would also depend on ADAM17. In fact, treatment of THP-1 cells with *E. coli* pHrodo induced the release of TNFα, which is one of the main substrates of ADAM17 [31]. Therefore, TNFα release by the phagocytosing THP-1 cells was reduced by knockdown of ADAM17, and to a lesser extent, by silencing of ADAM10 (Supplementary Figure S4C). The chemokine CXCL8, also termed interleukine-8 (IL-8), is known to be released by phagocytosing cells. Indeed, CXCL8 was released in our experiment, when THP-1 cells were treated with *E. coli* pHrodo. In contrast to TNFα, knockdown of ADAM17 further increased the CXCL8 release in *E.coli* pHrodo-treated THP-1 cells by nearly three-fold (Figure 4D). In line with the protein data, gene expression of CXCL8 in *E. coli* pHrodo treated THP-1 cells was also enhanced by ADAM17 knockdown, but not by knockdown of ADAM10 (Figure 4E). Yet, the effect at the mRNA level was not as high as that seen on the CXCL8 protein release. So far, the results indicate that ADAM17-deficient cells exhibit elevated phagocytosis and CXCL8 secretion.

### 2.5. Knockdown of ADAM17 Increases Phagocytosis in a Cell Autonomous Manner

**Figure 5:**
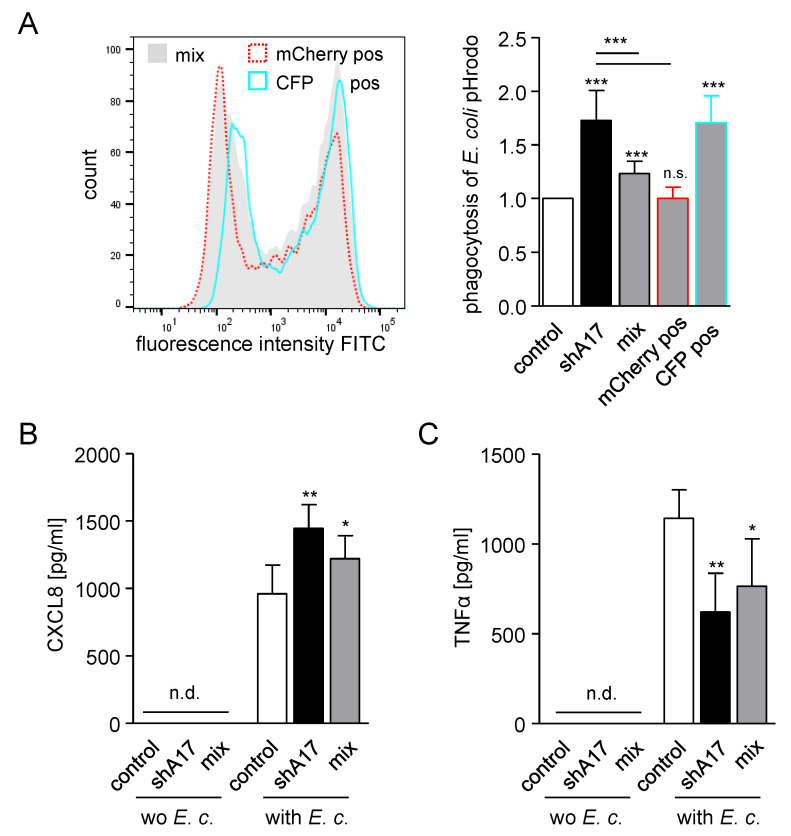
Knockdown of ADAM17 enhances phagocytosis in a cell autonomous manner, (**A**) THP-1 cells were transduced with lentivirus encoding control-shRNA (control, mCherry pos) or ADAM17-shRNA (shA17, CFP pos). A17 KD cells (black), control cells (white) and a 1:1 mixture of both (grey) were then exposed to *E. coli* pHrodo green and assayed for phagocytosis by flow cytometry. The geometric mean fluorescence of the cells was calculated in relation to that of the indicated control. Mixed cells were additionally gated for mCherry-positive (control–shRNA) or CFP-positive (ADAM17–shRNA) cells and reanalysed for their bacterial uptake. (**B**,**C**) Additionally, cells described in (**A**) were analysed for their CXCL8 (**B**) and TNFα (**C**) release by ELISA. Release could not be detected (n.d.) in the absence of *E.coli.* Data are shown as mean and SD of at least four independent experiments (**A**) *n* = 5; (**B**,**C**) *n* = 4). Significant differences compared to the respective control are indicated as asterisks (* *p* < 0.05, ** *p* < 0.01, *** *p* < 0.001) and additional comparisons are specified by bars.

We then asked whether the effect of ADAM17 on particle uptake and mediator release occurs in a cell autonomous manner. Therefore, THP-1 cells were transduced with either a mCherry-containing control or a CFP-containing ADAM17–shRNA vector. Subsequently, these cells were mixed in a 1:1 ratio and used in the phagocytosis assay. As shown beforehand, knockdown of ADAM17 led to enhanced uptake of *E. coli* and *S. aureus* pHrodo (Figure 5A; Supplementary Figure S5A). Mixed THP-1 cells showed an intermediate phagocytosis rate, which was still significantly increased compared to the control cells. Moreover, these cells were gated for mCherry-positive (control) or CFP-positive (ADAM17–shRNA) cells and reanalysed for their particle uptake. The mCherry positive cells revealed the same phagocytosis rate as the control cells, whereas the CFP positive cells showed a particle uptake comparable to that of the ADAM17 knockdown cells. From this experiment, it can be concluded that knockdown of ADAM17 increases bacterial phagocytosis in a cell autonomous manner.

Additionally, the release of CXCL8 and TNFα of these *E. coli* or *S. aureus* pHrodo treated cells was measured (Figure 5B,C; Supplementary Figure S5B,C). In accordance with our previous experiment (Figure 4C,D, Supplementary Figure S4C), presence of the pHrodo bacteria led to higher amounts of CXCL8 and TNFα in the supernatants. Whereas the release of CXCL8 was further increased, soluble TNFα was decreased by knockdown of ADAM17. Matching the phagocytosis data, mixed cells showed an intermediate release in both cases. These results also support our hypothesis, that enhanced phagocytosis due to knockdown of ADAM17 is accompanied by CXCL8 release and that the described effect occurs cell autonomously.

Since toll-like receptors (TLR) and scavenger receptors play a critical role in the binding and uptake of bacteria and are described for possible ADAM17 substrates [24,26], we analysed the surface expression of TLR2, TLR4, TLR6 and CD36 in transduced THP-1 cells after treatment with *E. coli* or *S. aureus* pHrodo (Supplementary Figure S6A–D). In addition, we checked the surface expression of TNFα-receptor types 1 and 2 (TNFR1/2), which are also shed by ADAM17 [32] (Supplementary Figure S6E,F). While the surface expression of TLR2, TLR4 and TNFR2 was not affected, treatment of the transduced THP-1 cells with either *E. coli* or *S. aureus* pHrodo led to a reduced TNFR1 expression ( Figure S6E). TNFR1 expression seemed to be slightly restored when ADAM17 was silenced but this effect was not significant. Furthermore, the surface expression of TLR6 was slightly increased (Supplementary Figure S6C), whereas the expression of CD36 was decreased ( Figure S6D) by ADAM17 knockdown in the bacteria treated THP-1 cells. Taken together, these results indicate that the enhanced phagocytosis in ADAM17 knockdown cells is associated with minimal changes in the surface expression of TNFR1, TLR6 and CD36 but not of TLR2, TLR4 and TNFR2.

### 2.6. Inhibition of TNFα Diminishes Phagocytosis and CXCL8 Release

**Figure 6:**
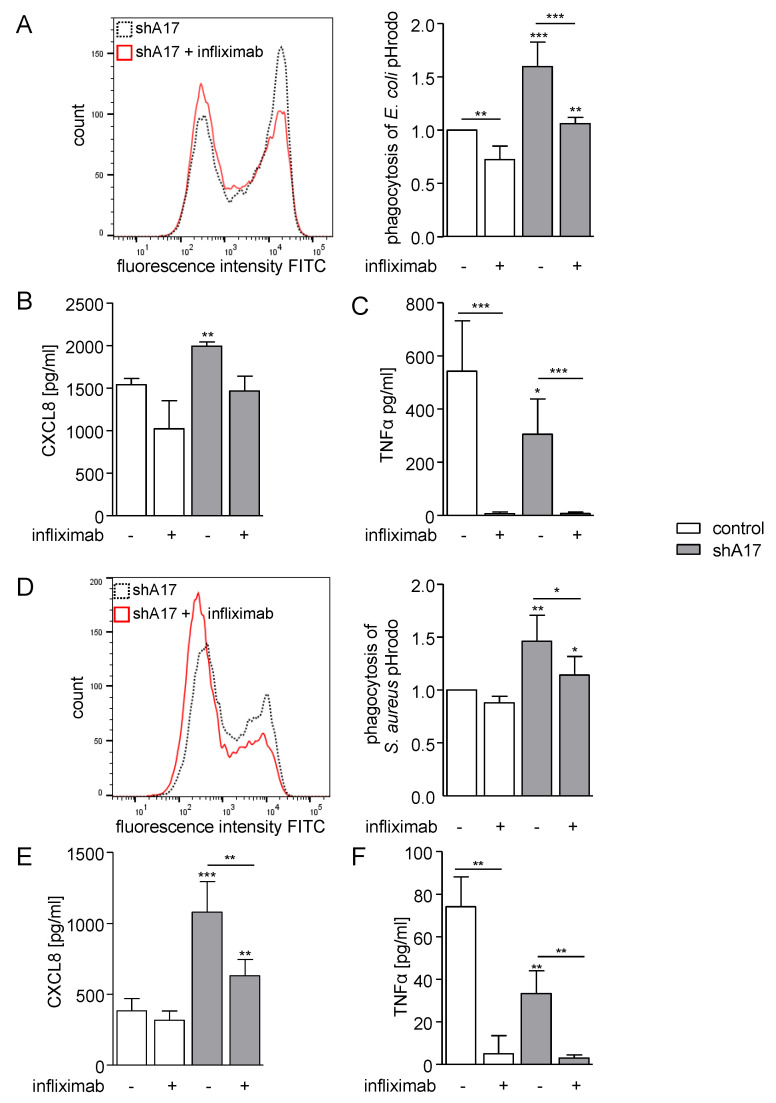
Inhibition of TNFα diminishes phagocytosis and CXCL8 release, (**A**–**F**) THP-1 cells were transduced with lentivirus encoding control–shRNA (control) or ADAM17–shRNA (shA17) and preincubated with 10 µg/mL infliximab or vehicle control. Subsequently, the transduced cells were incubated with *E. coli* (**A**–**C**) or *S. aureus* pHrodo green (**D**–**F**) and assayed for phagocytosis by flow cytometry (**A**,**D**) and for CXCL8 (**B**,**E**) and TNFα (**C**,**F**) release by ELISA. The geometric mean fluorescence of the cells was calculated in relation to that of the indicated control. Data are shown as mean and SD or representative histogram of at least three independent experiments (**A**,**D**) *n*=5; (**B**,**C**,**E**,**F**) *n* = 3. Significant differences compared to the respective control are indicated as asterisks (* *p* < 0.05, ** *p* < 0.01, *** *p* < 0.001) and additional comparisons are specified by bars.

To further study the potential involvement of TNFα, CXCL8 or growth factors of the EGF family in the phagocytic response we applied different pharmacological inhibitors. We used the inhibitory antibody infliximab which is capable of blocking membrane bound and soluble TNFα in its monomeric and biologically active trimeric state. Treatment with infliximab clearly reduced the phagocytosis of *E. coli* and *S. aureus* pHrodo (Figure 6A,D). Here, in wild type cells, a decrease in *E.coli* uptake was observed, which was more prominent in ADAM17-deficient cells (Figure 6A). In case of *S. aureus*, a significant reduction in particle uptake was only seen in cells with ADAM17 knockdown (Figure 6D). A contribution of TNFα signalling was also observed for CXCL8 release, which was studied in parallel. While the *E. coli*-induced response was similarly, but not significantly, reduced by infliximab in cells with or without ADAM17 knockdown (Figure 6B), *S. aureus* induced CXCL8 release was more sensitive to TNFα-inhibition when ADAM17 was silenced (Figure 6E). In parallel, we performed a retrieval analysis of TNFα and confirmed that infliximab efficiently neutralised the cytokine release in response to treatment with either bacterial strain (Figure 6C,F).

In addition, we used etanercept for TNFα inhibition and could confirm a role of endogenous TNFα induction and signalling for phagocytosis of *E.coli*, especially in cells with ADAM17 knockdown (Supplementary Figure S7A). Inhibition of CXCL8 signalling with the allosteric CXCR1 and two-antagonist reparixin or inhibition of EGFR signalling with the monoclonal antibody cetuximab seemed to have a minor effect on the phagocytosis of *E. coli* in those cells with ADAM17 knockdown (Supplementary Figure S7B,C). Finally, combinations of reparixin and/or cetuximab with etanercept did not yield additive effects on phagocytosis compared to etanercept alone. (Supplementary Figure S7D–F). These findings demonstrate that a lack of ADAM17 activity promotes bacterial uptake by phagocytes and this may involve several pathways, including TNFα-signalling.

## 3. Discussion

In the present study, we provide multiple lines of evidence that physiologic expression of ADAM17 in phagocytes attenuates bacterial uptake. This was shown not only for human and murine cell lines but also for primary cells including bone marrow-derived macrophages and neutrophils. Moreover, this effect was observed for a Gram-negative and for a Gram-positive bacterial species. Inhibition, knockdown and knockout approaches consistently demonstrate that lack of ADAM17 activity enhances bacterial phagocytosis by these cells. Interestingly, this enhanced bacterial uptake occurs in a cell autonomous manner and involves TNFα-signalling.

Previous studies demonstrated crucial functions of ADAM17 in immune defence. In particular, ADAM17 was found to promote bacterial sepsis in mice by shedding of TNFα [14]. The protease is also involved in IL6 trans-signalling by shedding of IL-6R, which in turn binds IL6, leading to a proinflammatory response. [33,34]. These effects are counteracted by shedding of TNFR1 which acts as a soluble TNFα antagonist [20]. Moreover, ADAM17 can suppress leukocyte recruitment to specific sites of inflammation by the shedding of L-selectin [21], which is required for the initial step of leukocyte transmigration through the endothelium. Our present study adds one more detail to the complex actions of ADAM17 during antibacterial defence by demonstrating that ADAM17 attenuates phagocytosis. In addition to ADAM17, ADAM10 is also involved in the regulation of the immune response, e.g., it sheds several adhesion molecules and is necessary for the migration of macrophages [33]. Although some substrates and activities are shared by the proteases, our results suggest that bacterial uptake is affected by ADAM17 but not by ADAM10. iRhoms have been discovered as critical adapter molecules required for maturation and subsequent activity of ADAM17 in vitro and in vivo [6,7,8]. In particular, leukocytes predominantly rely on iRhom2 to promote ADAM17 maturation. Deficiency of iRhom2 not only attenuates TNFα production by leukocytes but also reduces several leukocyte driven immune responses including bacterial sepsis and rheumatoid arthritis [7,10]. In line with this, and consistent with our own results regarding increased uptake of bacteria in case of ADAM17-knockout, we found that iRhom2-deficiency enhances phagocytosis. One would expect that iRhom1-deficiency does not have a similar effect, as it is expressed to a much lesser extent in phagocytes.

Previous studies have shown that activation of bacterial uptake by phagocytes is associated with an enhanced production of inflammatory mediators. This includes the release of several chemokines including CXCL8 [35,36,37]. This chemokine can serve to recruit further phagocytes via its receptors CXCR1 and 2. Of note, we found that lack of ADAM17 activity not only enhances bacterial uptake but also increases CXCL8 production. This increased CXCL8 release could then function to promote further recruitment of leukocytes and by this augment the immune response. However, we found that blocking of CXCR1 and two by reparixin does not have an influence on the phagocytosis of *E. coli* pHrodo. Therefore, the enhanced bacterial uptake in ADAM17-deficient cells cannot be explained by a feedback loop of increased CXCL8 release.

Interestingly, CXCL8 production in response to LPS by epithelial cells or smooth muscle cells can depend on activation of the EGFR via an autocrine mechanism involving shedding of EGFR ligands by ADAM17 [19,38]. In our experiments, however, a participation of the EGFR signalling pathway in particle uptake or CXCL8 production could be neither shown in ADAM17-expressing cells, nor in knockdown cells (CXCL8 data not shown).

One potential mechanism by which ADAM17 could affect bacterial uptake is via the release of PRRs. In fact, ADAM17 is known to shed several scavenger receptors involved in bacterial uptake including CD163, CD36 and CXCL16 [22,23,24,25]. Recent studies showed that ADAM17-deficiency improves recovery of mice after spinal cord injury. This was associated with increased uptake of apoptotic neuronal cells by microglia and increased CD36 expression on microglia. [30]. However, in our study we could not observe an increase in the CD36 level on the cell surface of monocytic cells after ADAM17 knockdown. Instead the expression was rather decreased, which may be due to increased internalisation of this scavenger receptor together with enhanced particle uptake in ADAM17 knockdown cells. Furthermore, it is uncertain whether the uptake of apoptotic cells by microglia cells can be compared with the uptake of bacteria by peripheral phagocytes.

We found that phagocytosis is upregulated only on those cells with knockdown of ADAM17 and that this effect cannot be transferred to co-cultured control cells. Therefore, a cell autonomous mechanism seems be responsible for the observed increase in the bacterial uptake. ADAM17-deficiency could lead to reduced shedding of a cell-expressed substrate and the resulting increased surface expression of the substrate could thus stimulate bacterial uptake. Of note, pattern recognition receptors such as TLR2 and TLR4 can undergo ADAM17-mediated shedding, which could lead to decreased stimulation of phagocytosis by bacterial components [26,39]. However, TLR2 and TLR4 surface expression was not affected by ADAM17 knockdown under our experimental conditions. Interestingly, a higher TLR6 expression was observed in phagocytosing THP1 cells with ADAM17 knockdown. Since TLR6 is involved in recognition of diacyl lipopeptides its enhanced surface expression could be involved in the increased uptake of Gram-positive bacteria.

In our inhibition experiments, we addressed the question whether autocrine stimulation via cell-expressed receptors is altered upon ADAM17 knockdown. Inhibition with infliximab and etanercept indicates that TNFα-signalling in monocytic THP-1 cells enhances bacterial uptake. Strikingly, the observed inhibition was negatively proportional to the level of ADAM17 activity. These findings indicate that enhanced phagocytosis upon ADAM17 knockdown depends at least in part on TNFα. On the one hand, this was surprising since ADAM17 downregulation reduces TNFα-release by phagocytosing cells and it is unclear how the reduced TNFα-release is related to enhanced phagocytosis upon ADAM17-deficiency. On the other hand, this deficiency will increase the surface level of transmembrane TNFα. Moreover, ADAM17-downregulation could reduce shedding of the receptor TNFR1. This could lead to enhanced cell-to-cell stimulation via more transmembrane TNFα-binding to more cell expressed TNFR1. However, we did not see a prominent increase in TNFR1 expression in cells lacking ADAM17 activity under our experimental conditions. Therefore, the molecular details of the mechanism involving TNFα are not clear yet.

Our results could be of relevance for the modulation of ADAM17 activity by endogenous mechanisms. Tissue inhibitor of metalloproteinase 3 (TIMP3) is a potent and physiologically relevant endogenous inhibitor of ADAM17 [40]. Further studies need to clarify whether endogenous ADAM17 inhibition by TIMP3 is associated with increased bacterial uptake and improved anti-infectious defence. Vice versa, it will be interesting to study whether endogenous upregulation of ADAM17 activity would lead to a stronger suppression of bacterial uptake. Several mechanisms of activity upregulation are known. Recent studies indicate that upregulation of only ADAM17 protein synthesis may be insufficient [41]. This might be due to the fact that ADAM17-activity is controlled by other mechanisms, including ADAM17 trafficking by iRhoms and conformational changes [8,42]. Whether these mechanisms promoting enhanced ADAM17 activity also lead to suppression of phagocytosis and potential immune deficiencies needs to be studied.

Since the discovery of ADAM17 as a shedding enzyme for TNFα, it is considered as a target for treating TNFα-mediated diseases. In the case of ADAM17 inhibition, however, side effects must be expected due to the involvement of the protease in a large number of signalling pathways. As mentioned before, ADAM17 inhibition or knockout improved the uptake of apoptotic cells by microglia after spinal cord injury in mice [30]. Whether treatment with an ADAM17 inhibitor would also improve bacterial uptake by either microglial cells or potentially also by peripheral phagocytes and thereby improved antibacterial defence needs to be further studied in murine models of infection.

## 4. Materials and Methods

### 4.1. Antibodies, Kits and Reagents

Antibodies, kits and reagents are listed in the Supplemental Materials.

### 4.2. Cell Culture and Transduction

The human monocytic leukemia cell line THP-1 was cultured in RPMI medium with 10% fetal bovine serum (FBS) (PanBiotech, Aidenbach, Germany) and 1% Penicillin-Streptomycin (Sigma-Aldrich, St. Louis, MO, USA), by seeding 5 × 10^5^ cells/mL and subculture at 1 × 10^6^ cells/mL. The murine macrophage cell line RAW264.7 was cultured in DMEM with 10% FBS and 1% Penicillin-Streptomycin. Human neutrophils and human peripheral blood mononuclear cells (PBMC) from citrated (0.38%) peripheral blood of healthy volunteers and murine bone marrow-derived macrophages (BMDM) out of the femur and tibia of hind limbs of 8 to 12 week old mice were isolated and cultured as described [43,44]. BMDMs were either generated out of total *iRhom2* KO (heterozygous or homozygous) mice (generated with CRISPR/Cas9 technology) and their respective littermates, or out of Vav guanine nucleotide exchange factor 1 (Vav)-*Adam10*^−/−^ mice and Vav-*Adam17*^−/−^ mice that expressed *Cre* recombinase under control of the Vav promotor and were homozygous for floxed *Adam10* or floxed *Adam17*, respectively. Littermate controls of the same background carried *Cre* recombinase but no floxed protease genes, resulting in wild type expression of ADAM10 and ADAM17.

Short hairpin RNA (shRNA) targeting ADAM10 (5′ CGC GTC CCC ACA GTG CAG TCC AAG TCA ATT CAA GAG ATT GAC TTG GAC TGC ACT GTT TTT TGG AAA T 3′) or ADAM17 (5′ CGC GTC CCC AGG AAA GCC CTG TAC AGT ATT CAA GAG ATA CTG TAC AGG GCT TTC CTT TTT TGG AAA T 3′) was inserted into the lentiviral expression vector pLVTHM-CFP and shRNA targeting iRhom2 (GTG AAG CAC TTT GCC TTT GAT CTC GAG ATC AAA GGC AAA GTG CTT CAC TTT TTG) was inserted into the lentiviral expression vector pLKO.1 as described [45,46]. Inserted irrelevant shRNA (pLVTHM-CFP/mCherry; 5′ CGC GTC CCC ACC GTC TGT GTA TCG TCG CTT CAA GAG AGC GAC GAT ACA CAG ACG GTT TTT TGG AAA T 3′) and pLKO.1 non-mammalian shRNA control plasmid DNA (SHC002, Merck, Darmstadt, Germany) served as a control. For transduction, 2 × 10^5^ THP-1 cells were seeded into 12-wells and concentrated lentivirus preparation (5 µL) was added. To enhance the transduction efficiency polybrene (4 µg/mL, Sigma-Aldrich, St. Louis, MO, USA) was used.

### 4.3. Cell Treatment and Phagocytosis Assay

For phagocytosis assays 5 × 10^5^ THP-1 cells/mL, 2 × 10^5^ RAW264.7 cells/mL or 2 × 10^5^ BMDMs/mL were seeded in fully supplemented growth medium 24 h before bacterial treatment. Isolated human peripheral blood mononuclear cell (PBMCs) and neutrophils were directly seeded in round bottom tubes (5 × 10^5^/mL) and incubated for 2 h at 37 °C prior infection. If an inhibitor was used, 10 µM TAPI-1, 10 µg/mL etanercept (enbrel^®^), 10 µg/mL infliximab, 10 µM reparixin, 1 µg/mL cetuximab or 0.1% dimethylsulfoxide (DMSO) (TAPI-1 control) or 0.9% NaCl as vehicle control was added 30–45 min before treatment with *E. coli/S. aureus* pHrodo green. Phagocytosis was analysed after 1 h (RAW264.7, PBMCs, neutrophils, BMDMs) or 4 h (THP-1). Therefore, cells were washed with PBS and adherent cells (RAW264.7, BMDMs) were harvested by usage of a rubber policeman (VWR, Langenfeld, Germany). The fluorescence signal of control cells (without bacteria) was subtracted from the corresponding samples. If BMDMs were exposed to *E. coli* GFP, the medium was replaced by antibiotic-free RPMI supplemented with 10% FBS. *E. coli* GFP bacteria were freshly grown in Lennox-L-Broth (LB)-medium with 0.2 mM isopropyl β-D-1-thiogalactopyranoside (IPTG) to initiate GFP expression until early logarithmic growth, resuspended in the respective antibiotic-free medium supplemented with 10% FBS and used immediately. Infection was performed at a multiplicity of infection (MOI) of 25 or 50. To exclude bacteria that were not internalized but just bound to the cells, 0.4% trypan blue, which in direct contact quenches the GFP/FITC signal, was added to the cells directly before they were analysed by flow cytometry. In addition, 4 °C controls were made and their fluorescence signal was subtracted from the respective 37 °C samples.

### 4.4. Flow Cytometric Analysis

PBS supplemented with 1% FBS (PanBiotech, Aidenbach, Germany) and 1 mM EDTA was used as assay buffer for murine cells and PBS supplemented with 0.2% BSA was used as assay buffer for human cells. The whole staining process was performed at 4 °C. Cells were stained with the respective antibody for 45 min on ice (for antibody information see Supplemental Materials and figure legends). After washing and resolving in PBS, the fluorescence signal of stained THP-1 cells, BMDMs or cells from phagocytosis assays was analysed by flow cytometry (LSRFortessa, BD Biosciences, Heidelberg, Germany) and evaluated with FlowJo V10 software (Ashland, USA).

### 4.5. Cell Lysis and Western Blotting

For analysis of cellular proteins cell lysates were generated using a lysis buffer containing 20 mM Tris-HCl, 150 mM NaCl, 1% TritonX-100, 5 mM EDTA, 1 mM PMSF, 10 mM 1,10-phenanthroline monohydrate, one-fold Complete (Roche) and 10 µM GI254023X. After 20 min incubation on ice, lysates were cleared by 10 min centrifugation at 16,100× *g* and protein content was analysed via a BCA assay (Interchim, Montluçon, France) according to manufacturer’s instructions.

Samples from cell lysates (20 µg total protein), were heated in reducing SDS sample buffer (250 mM Tris HCl (pH 6.8), 50% (*w*/*v*) glycerol, 10% (*w*/*v*) SDS, 0.1% bromophenol blue, 5% β-mercaptoethanol) and subjected to SDS-polyacrylamide gel electrophoresis using 10% Tris-glycine gels. Proteins were transferred onto polyvinylidene difluoride membranes (Hybond-P, Amersham). Membranes were blocked with 5% (*w*/*v*) non-fat dry milk in TBS with 0.05% Tween for 1 h and probed with primary antibodies for 1 h at room temperature or overnight at 4 °C followed by incubation with HRP-coupled secondary antibodies (diluted 1:30,000) for 1 h. After addition of enhanced chemiluminescence substrate (ECL Prime, GE Healthcare), signals were recorded using the LAS 3000 Image Analyzer^®^ (Fujifilm, Tokyo, Japan).

### 4.6. ELISA

Release of hCXCL8 and hTNFα to the supernatant of stimulated THP-1 cells was analysed using commercial ELISA Kits (DuoSet, R&D Systems, Minneapolis, MN, USA) according to the manufacturer’s protocols. The read-out at 450 nm and 540 nm was executed with a FLUOstar OPTIMA (BMG-Labtech, Ortenberg, Germany).

### 4.7. Quantitative PCR Analysis

The mRNA expression level of ADAM10, ADAM17, iRhom2 and CXCL8 was measured by quantitative real-time PCR and normalized to the mRNA expression level of glyceraldehyde-3-phosphate dehydrogenase (*GAPDH*, human samples) or *Gapdh* and *Rps29* (murine samples). mRNA was extracted using RNeasy Kit (Qiagen, Hilden, Germany) and quantified photometrically (NanoDrop, Peqlab, Erlangen, Germany). 200–300 µg RNA was reverse transcribed using PrimeScript™ RT Reagent Kit (Takara Bio Europe, St-Germain-en-Laye, France) and PCR reactions were performed using SYBR Premix Ex Taq II (Takara Bio Europe) or iTaq™ Universal SYBR^®^ Green Supermix (Bio Rad, Hercules, CA, USA) according to the manufacturer’s protocols. The primers used and their corresponding annealing temperature can be found in the Supplemental material (Supplementary Table S1). PCR reactions were run on a LightCycler 480 System (Roche, Basel, Switzerland) or a CFX Connect Real-Time PCR Detection System (Bio-Rad, Hercules, CA, USA) with 45 cycles of 10 s denaturation at 95 °C, followed by 30 s annealing and 15 s amplification at 72 °C. For qPCR runs carried out on the Bio-Rad system the efficiency of each qPCR sample was calculated with linear regression using the LinReg software (v2018.0, Dr. J.M. Ruitjer, Academical Medical Centre Amsterdam, The Netherlands). If qPCR was performed on the Roche System the efficiency of each qPCR run was calculated with standard curves, which were determined by serial dilution of a defined cDNA standard within each data set. Relative quantification was performed with the E-Method (Tellmann, 2006) using the LightCycler^®^480 software (Roche, Basel, Switzerland) or the CFX Maestro 1.1 software (v4.1.2433.1219, Biorad, Hercules, CA, USA).

### 4.8. Statistics

Quantitative data are shown as mean and standard deviation (SD) calculated from at least three independent experiments. For a better graphical presentation and comparison flow cytometry data were normalized by expression in relation to the appropriate controls. Raw data were analysed by general mixed model analysis (PROC GLIMMIX, SAS 9.4, SAS Institute Inc., Cary, NC, USA) and assumed to be derived from either log normal or exponential distributions; residual plots and the Shapiro Wilk test were used as diagnostics. If necessary, the day of experiment conduction was set as random to assess differences in the size of treatment effects across the results. According to the covtest statement, all data sets were homoscedastic. Multiple comparisons were corrected by false discovery rate (FDR).

## Supplementary Materials

Supplementary materials can be found at https://www.mdpi.com/1422-0067/21/17/5978/s1.

## Abbreviations

ADAM	a disintegrin and metalloproteinase
BMDM	bone marrow-derived macrophage
ctrl	control
CFP	cyan fluorescent protein
CXCL8	C-X-C motif chemokine ligand 8
DMEM	Dulbecco’s Modified Eagle’s Medium
*E. coli (E. c.)*	*Escherichia coli*
EGF	epidermal growth factor
EGFR	epidermal growth factor receptor
ER	endoplasmic reticulum
FBS	fetal bovine serum
FDR	false discovery rate
GAPDH	glyceraldehyde 3-phosphate dehydrogenase
GFP	green fluorescent protein
IL-6R	Interleukin-6 receptor
JAM-A	junctional adhesion molecule
KO	knockout
LPS	lipopolysaccharide
MPD	membrane-proximal domain
n.d.	not detected
*S. aureus (S. a.)*	*Staphylococcus aureus*
PBMC	peripheral blood mononuclear cell
PRR	pattern recognition receptor
shRNA	small hairpin RNA
TAPI	TAPI-1
TIMP3	tissue inhibitor of metalloproteinase 3
TLR	toll-like receptor
TNFα	tumour necrosis factor-α
TNFR	TNF receptor
WT	wild type
