# Supplementary material for: The iRhom2/ADAM17 Axis Attenuates Bacterial Uptake by Phagocytes in a Cell Autonomous Manner
Source: Int J Mol Sci. 2020 Aug 19;21(17):5978. doi: 10.3390/ijms21175978 (PMC7503280; doi:10.3390/ijms21175978)

Supplements

Supplemental Materials

Antibodies, kits and reagents

Mouse monoclonal antibody against human/murine GAPDH (MA5-15738), rat monoclonal eFluor 450-conjugated CD11b antibody (48-0112-82) and mouse monoclonal APC-conjugated human TLR2 (17-9922-42) and TLR4 (17-9917) antibodies, were from Thermo Fisher Scientific (Waltham, USA). Mouse monoclonal antibody against human/mouse Transferrin receptor (ab1086) and rabbit polyclonal antibody against human/murine ADAM17 (ab39162) were from Abcam (Cambridge, UK). Rabbit polyclonal antibody against human/murine iRhom2 (SAB1304414) was from Sigma Aldrich (St. Louis, USA). Horse radish peroxidase (HRP)-conjugated goat anti-mouse and goat anti-rabbit antibodies were from Jackson ImmunoResearch Laboratories, Inc. (West Grove, USA). Rabbit polyclonal antibodies against the C-terminus of ADAM17 (AB19027) and ADAM10 (AB19026) were from Merck Millipore (Darmstadt, Germany). FITC-conjugated rat monoclonal anti-mouse F4/80 antibody (MCA497F) was from Serotec (Bio-Rad, Hercules, USA). Human CXCL8 (DY208) and TNFα (DY210) DuoSet ELISA and mouse monoclonal APC-conjugated antibodies against human TNFR1 (FAB225A) and TNFR2 (FAB226A) were from R&D Systems (Minneapolis, USA). Rabbit polyclonal antibody against human TLR6 (AP1506A) was from Abgent (San Diego, USA) and its corresponding Alexa Fluor 555-conjugated secondary antibody (A-21428) was from Thermo Fisher Scientific. Mouse monoclonal APC-conjugated antibody against human CD36 (550956) was from BD (Franklin Lakes, USA). The metalloproteinase inhibitor TAPI-1 was from Merck (Darmstadt, Germany). GI254023X was synthesized and characterized as preferential ADAM10 inhibitor as previously reported [1,2]. Heat-inactivated *E. coli* pHrodo green (P35366) and *S. aureus* pHrodo green (P35382) were from Thermo Fisher Scientific (Waltham, USA). *E. coli* DH5α, an encapsulated K12 laboratory strain, carrying the green fluorescent protein (gfp)-mut2 gene (*E. coli* GFP) was generated by Stephan Dreschers (Department of Neonatology, University Aachen) [3]. LPS from *E. coli* strain 0127:B8 was from Sigma-Aldrich (St. Louis, USA). The TNFα-blocker etanercept (enbrel®) was from Wyeth Europa Ltd (South Maidenhead, UK) and infliximab was from Sigma-Aldrich (St. Louis, USA). The CXCR1/CXCR2 antagonist reparixin was from Tocris Bioscience (Bristol, UK) and cetuximab, an antibody against EGFR, was from Merck (Darmstadt, Germany).

Quantitative PCR analysis

Supplemental table 1:

gene	*primer*	*annealing temperature*
ADAM10 forward	ggattgtggctcattggtgggca	61 °C
ADAM10 reverse	actctctcggggccgctgac	
ADAM17 forward	aaacgtcatccggaggtcgcggcgccagcacgaagt	55 °C
ADAM17 reverse	taaaacttcgtgctggcgccgcgacctccggatgac	
RHBDF2 forward	cgattgacctgatccacc	58 °C
RHBDF2 reverse	caaagtctccgagcagtcc	
CXCL8 forward	tgcagctctgtgtgaaggtgcag	61 °C
CXCL8 reverse	tgtgttggcgcagtgtggtcc	
GAPDH forward	ccagccccagcgtcaaaggt	66 °C
GAPDH reverse	cggggctctccagaacatcatcc	
Adam10 forward	agcaacatctggggacaaac	57 °C
Adam10 reverse	tggccagattcaacaaaaca	
Adam17 forward	aaaccagaacagacccaacg	57 °C
Adam17 reverse	gtacgtcgatgcagagcaaa	
Rhbdf2 forward	agagcgtgaagtacatcc	60 °C
Rhbdf2 reverse	taaagtctccgagcagtcc	
Gapdh forward	ggcaaattcaacggcacagt	63 °C
Gapdh reverse	agatggtgatgggcttccc	
Rps29 forward	cctttctcctcgttgggc	61 °C
Rps29 reverse	gagcagacgcggcaa	

Supplemental references

1. Hundhausen, C.; Misztela, D.; Berkhout, T.A.; Broadway, N.; Saftig, P.; Reiss, K.; Hartmann, D.; Fahrenholz, F.; Postina, R.; Matthews, V.; et al. The disintegrin-like metalloproteinase ADAM10 is involved in constitutive cleavage of CX3CL1 (fractalkine) and regulates CX3CL1-mediated cell-cell adhesion. *Blood* **2003**, doi:10.1182/blood-2002-12-3775.

2. Ludwig, A.; Hundhausen, C.; Lambert, M.; Broadway, N.; Andrews, R.; Bickett, D.; Leesnitzer, M.; Becherer, J. Metalloproteinase Inhibitors for the Disintegrin-Like Metalloproteinases ADAM10 and ADAM17 that Differentially Block Constitutive and Phorbol Ester-Inducible Shedding of Cell Surface Molecules. *Comb. Chem. High Throughput Screen.* **2005**, doi:10.2174/1386207053258488.

3. Dreschers, S.; Platen, C.; Ludwig, A.; Gille, C.; Köstlin, N.; Orlikowsky, T.W. Metalloproteinases TACE and MMP-9 differentially regulate death factors on adult and neonatal monocytes after infection with Escherichia coli. *Int. J. Mol. Sci.* **2019**, doi:10.3390/ijms20061399.

Supplemental figure legends

Supplemental figure 1:

A) Representative image of *E. coli* pHrodo green particles phagocytosed by THP-1 cells (10 x objective). **B**) Representative dot plot diagrams showing FITC-positive gating of *E. coli* pHrodo treated THP-1 cells (compare Fig. 1A). **C**) THP-1 cells, human PBMCs, human neutrophils, murine RAW264.7 cells and murine BMDMs were preincubated with 10 µM TAPI or 0.1 % DMSO as vehicle control (ctrl), treated with *S. aureus* pHrodo green and assayed for phagocytosis by flow cytometry. The geometric mean fluorescence of the cells was calculated in relation to that of the respective control and summarized as mean and SD of at least four independent experiments (THP-1 n=8, PBMC and neutrophils n=4, RAW264.7 n=5, BMDM n=8). Significant differences compared to the respective control are indicated as asterisks (* $p < 0.05$, ** $p < 0.01$, *** $p < 0.001$).

Supplemental figure 2:

A) BMDMs were analysed for surface expression of the macrophage markers F4/80 and CD11b by antibody labelling and subsequent flow cytometry. **B**) Representative image showing *E. coli* GFP particles phagocytosed by murine BMDMs (20 x objective). **C, F**) Murine BMDMs of *VavCre Adam10* KO mice ($A10^{-/-}$) (C) or *VavCre Adam17* KO mice ($A17^{-/-}$) and their respective wild type littermates ($A10^{+/+}$, $A17^{+/+}$) were analysed for mRNA expression of *Adam10* (C) or *Adam17* (F) by RT-qPCR. **D**, **G**) Representative histograms comparing phagocytosis of *E. coli* GFP by ADAM10-deficient (D) or ADAM17-deficient (G) and wild type BMDMs (compare Fig. 2D, F). **E**, **H**) BMDMs as described in C and F were exposed to *S. aureus* pHrodo green and analysed for phagocytosis by flow cytometry. The geometric mean fluorescence of the cells was calculated in relation to that of the respective control. Data are shown as mean and SD of at least four independent experiments. (C n=5, E n=4, F n=7, H n=8). Significant differences compared to the respective control are indicated as asterisks (* $p < 0.05$, ** $p < 0.01$, *** $p < 0.001$).

Supplemental figure 3:

A) BMDMs of heterozygous ($iR2^{+/-}$) and homozygous *iRhom2* KO mice ($iR2^{-/-}$) and their respective wild type littermates were analysed for their *iRhom2* expression by RT-qPCR. **B-C**) THP-1 cells were transduced with lentivirus encoding control-shRNA (ctrl) or iRhom2-shRNA (iR2) and analysed for iRhom2 knockdown by RT-qPCR (B) or were exposed to *E. coli* pHrodo green and assayed for phagocytosis by flow cytometry (C). Data are shown as mean and SD or representative histogram of three independent experiments. Significant differences compared to the respective control are indicated as asterisks (* $p < 0.05$, ** $p < 0.01$, *** $p < 0.001$).

Supplemental figure 4:

A-C) THP-1 cells were transduced with lentivirus encoding control-shRNA (control), ADAM10-shRNA (shA10) or ADAM17-shRNA (shA17). Cells were analysed for ADAM10 (A) or ADAM17 (B) knockdown by RT-qPCR or were exposed to *E. coli* pHrodo green and assayed for TNFα release by ELISA. Data are shown as mean and SD of at least three independent experiments (A, B n=6, C n=3). Significant differences compared to the respective control are indicated as asterisks (* $p < 0.05$, ** $p < 0.01$, *** $p < 0.001$) and additional comparisons are specified by bars.

Supplemental figure 5:

A) THP-1 cells were transduced with lentivirus encoding control-shRNA (mCherry pos) or ADAM17-shRNA (CFP pos). A17 KD cells (black), control cells (white) and a 1:1 mixture of both (grey) were then treated with *S. aureus* pHrodo green and assayed for phagocytosis by flow cytometry. The geometric mean fluorescence of the cells was calculated in relation to that of the indicated control. Mixed cells were additionally gated for mCherry positive (control-shRNA) or CFP positive (ADAM17-shRNA) cells and reanalysed for their particle uptake. Additionally cells described in A were analysed for their CXCL8 (B) and TNFα (C) release by ELISA. Data are shown as mean and SD or as one representative histogram of at least four independent experiments (A n=5, B, C n=4). Significant differences compared to the respective control are indicated as asterisks (* $p < 0.05$, ** $p < 0.01$, *** $p < 0.001$) and additional comparisons are specified by bars.

Supplemental figure 6:

A-F) THP-1 cells were transduced with lentivirus encoding control-shRNA (ctrl) or ADAM17-shRNA (shA17), treated with *E. coli* pHrodo green, *S. aureus* pHrodo green or PBS and assayed for surface expression of TLR2 (A), TLR4 (B), TLR6 (C), CD36 (D), TNFR1 (E) and TNFR2 (F) by flow cytometry. Data are shown as mean and SD of three independent experiments. Significant differences compared to the respective control are indicated as asterisks (* $p < 0.05$, ** $p < 0.01$, *** $p < 0.001$) and additional comparisons are specified by bars.

Supplemental figure 7:

A-F) THP-1 cells were transduced with lentivirus encoding control-shRNA or ADAM17-shRNA and incubated with 10 µg/ml etanercept (A), 10 µM reparixin (B), 1 µg/ml cetuximab (C), a combination of etanercept and reparixin (D), etanercept and cetuximab (E), or etanercept, reparixin and cetuximab (F) or vehicle control. Subsequently, cells were exposed to *E. coli* pHrodo green and assayed for phagocytosis by flow cytometry. The geometric mean fluorescence was calculated in relation to the indicated control. Data are shown as mean and SD of five independent experiments. Significant differences compared to the respective control are indicated as asterisks (* $p < 0.05$, ** $p < 0.01$, *** $p < 0.001$) and additional comparisons are specified by bars.

Supplemental figure 1:

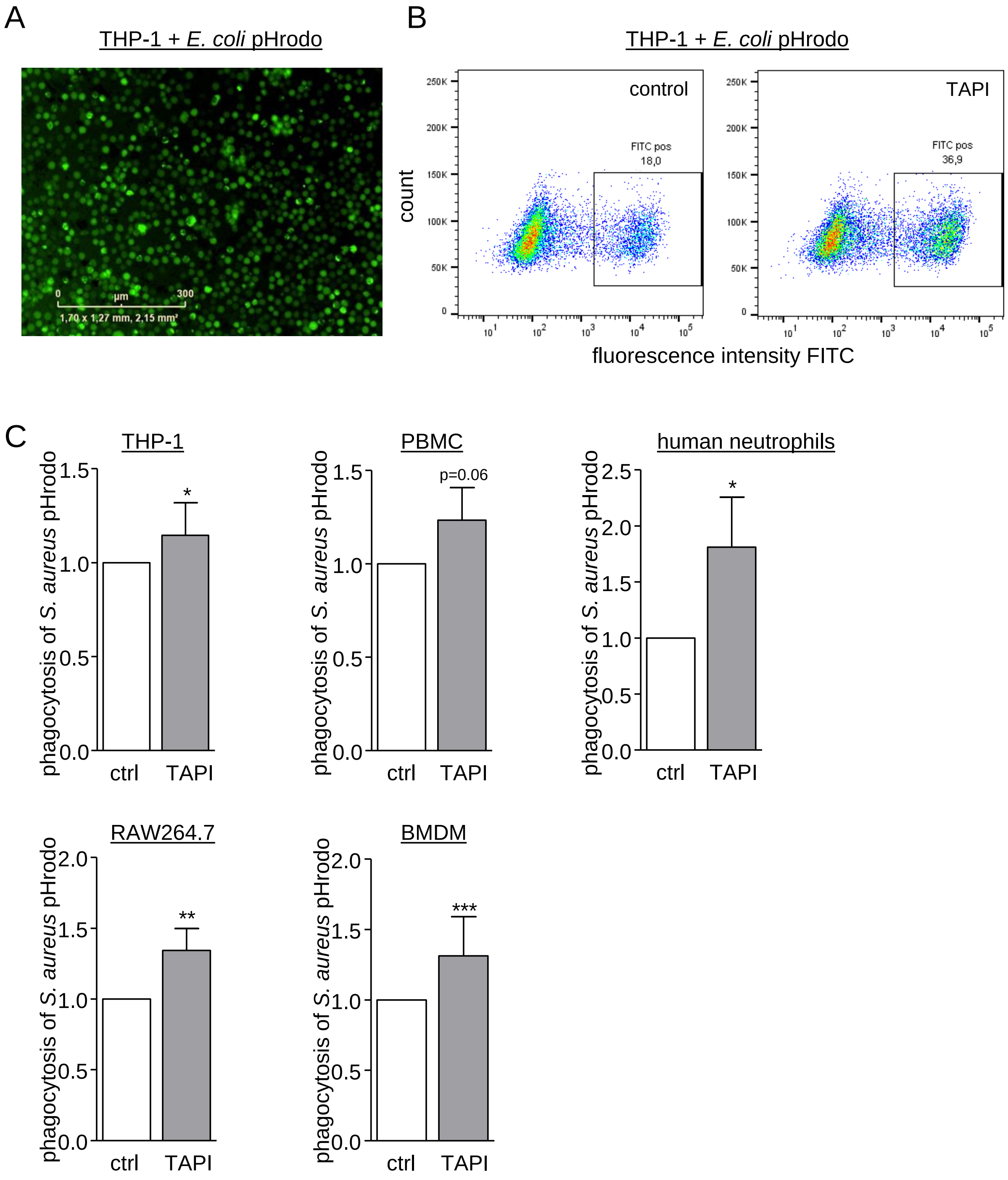

Supplemental figure 2:

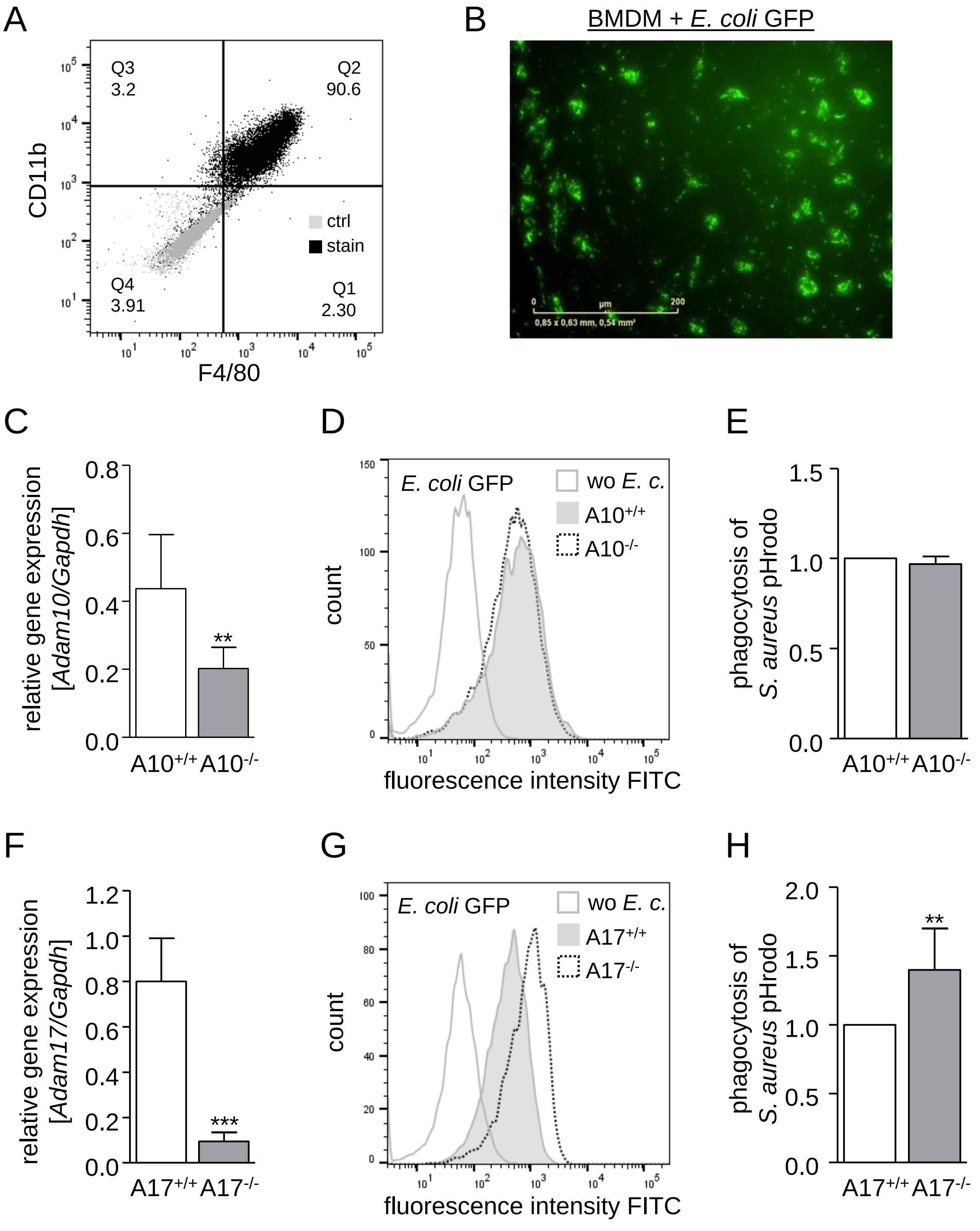

Supplemental figure 3:

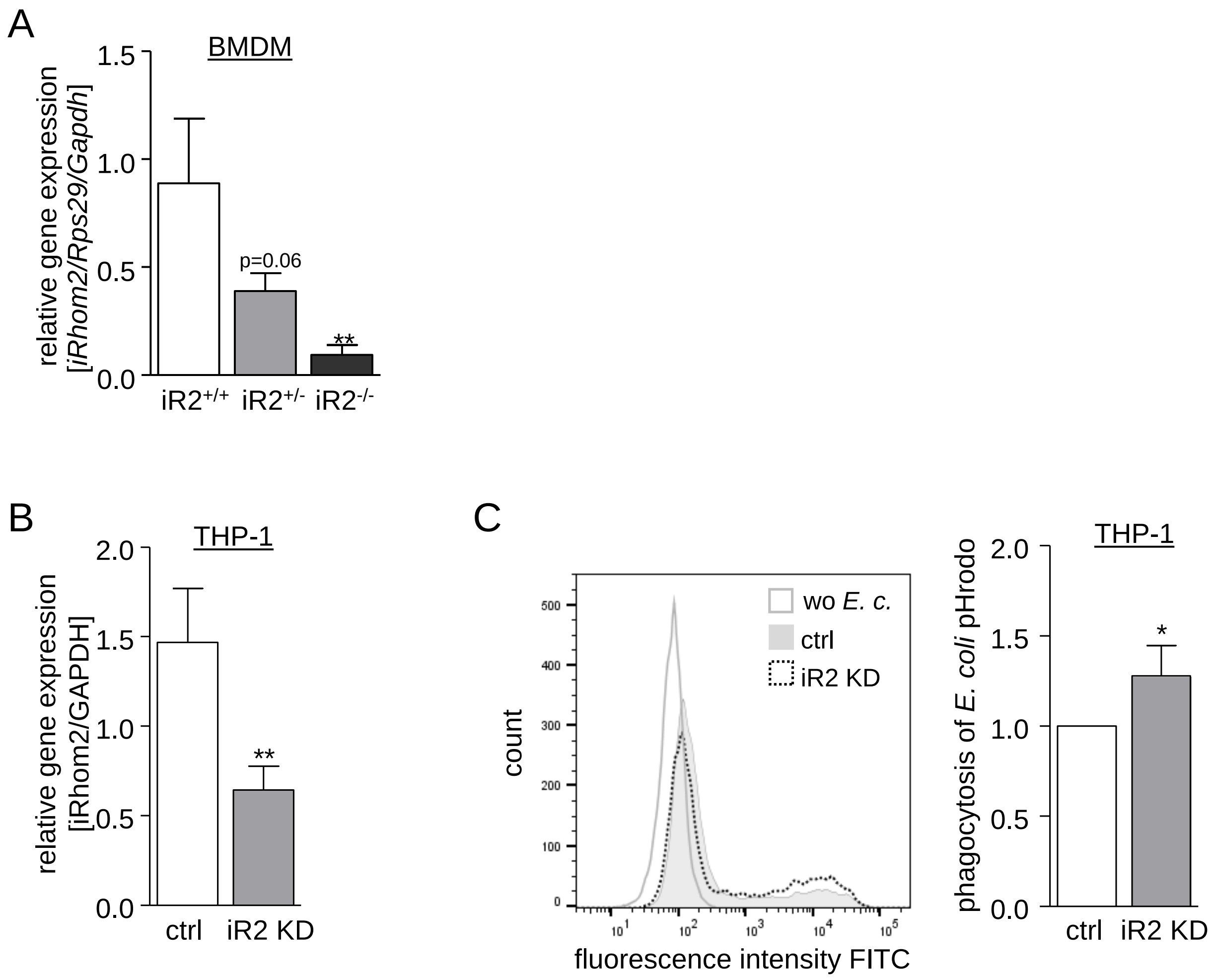

Supplemental figure 4:

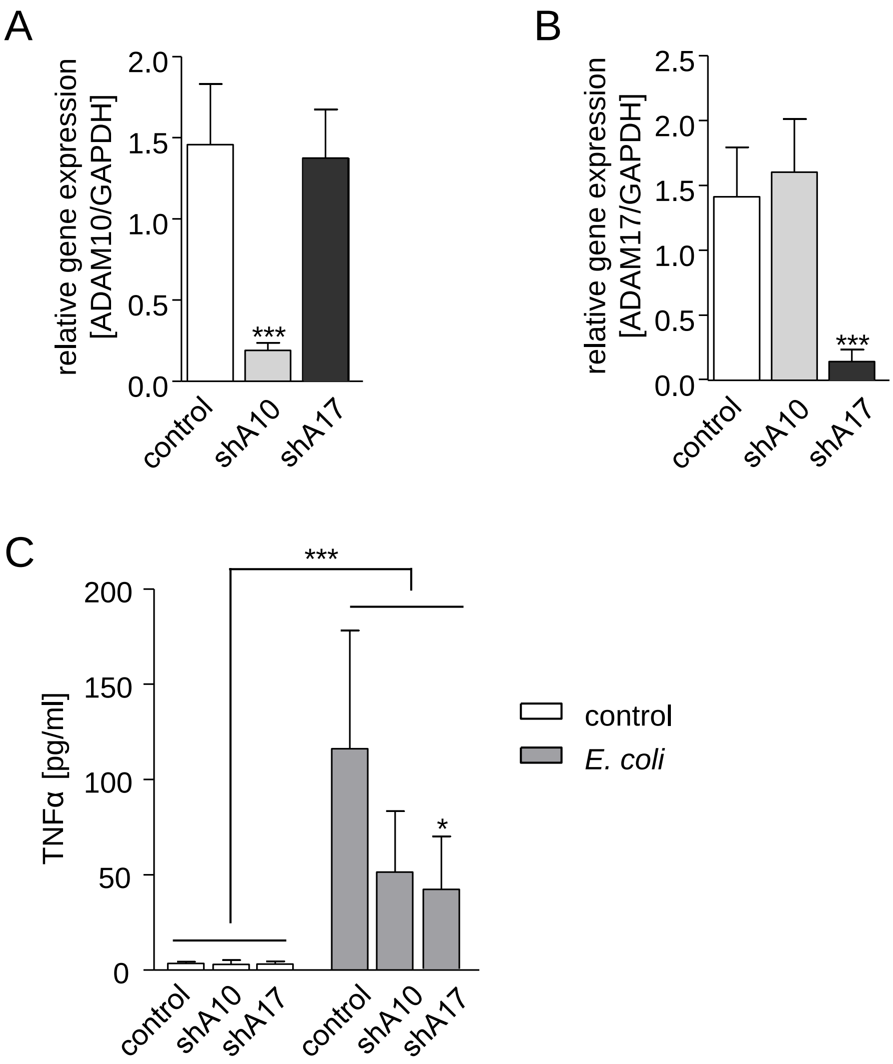

Supplemental figure 5:

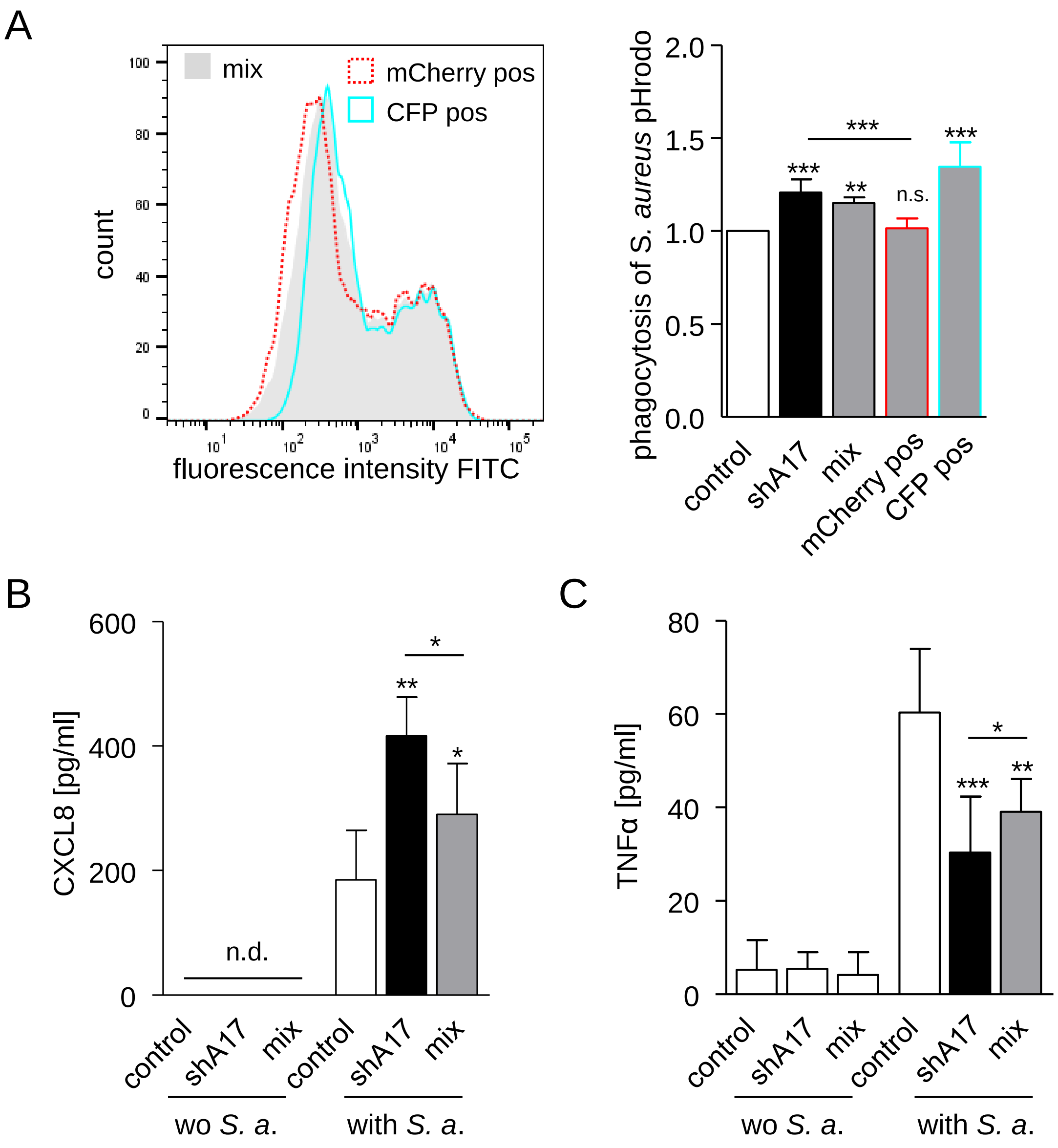

Supplemental figure 6:

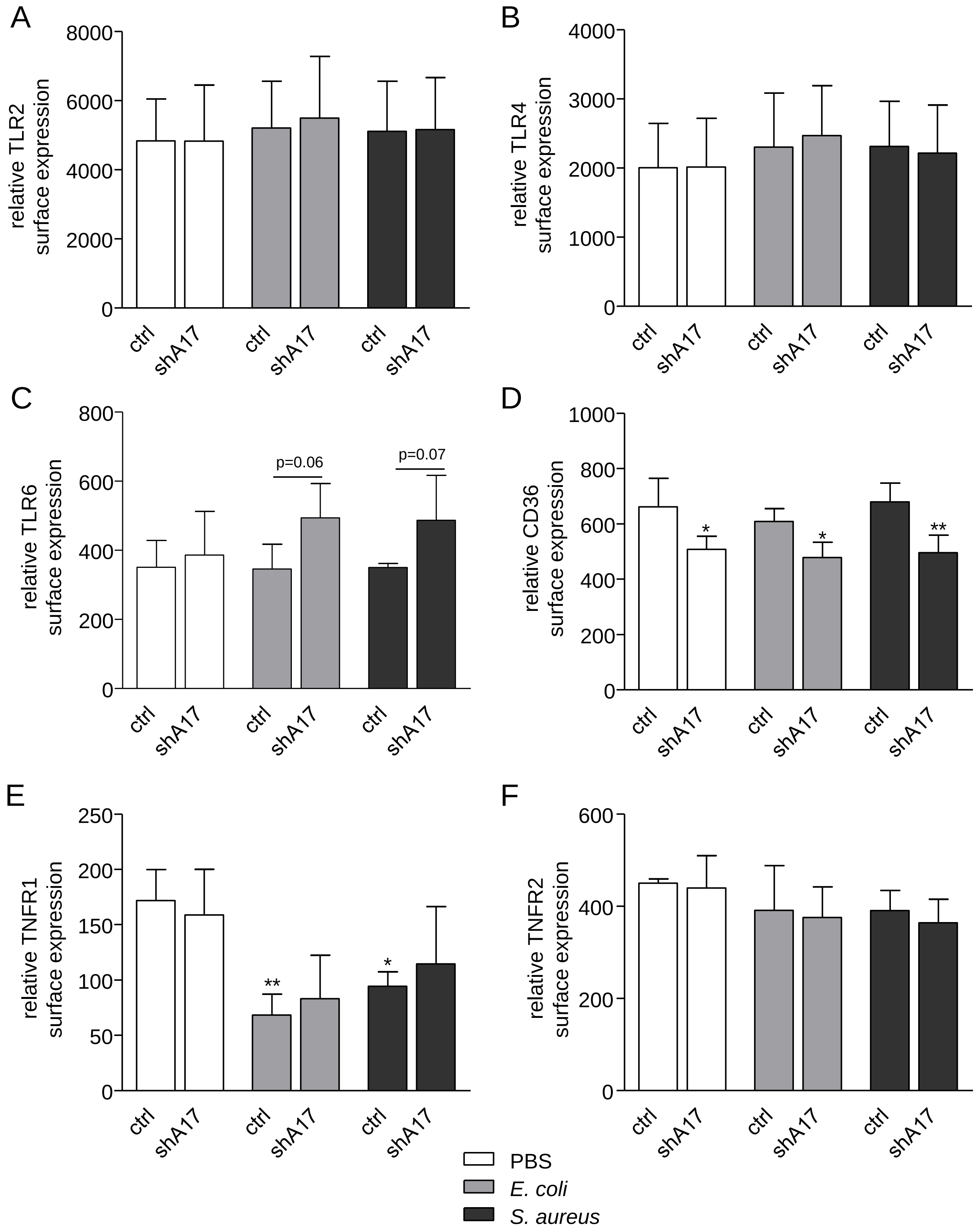

Supplemental figure 7:

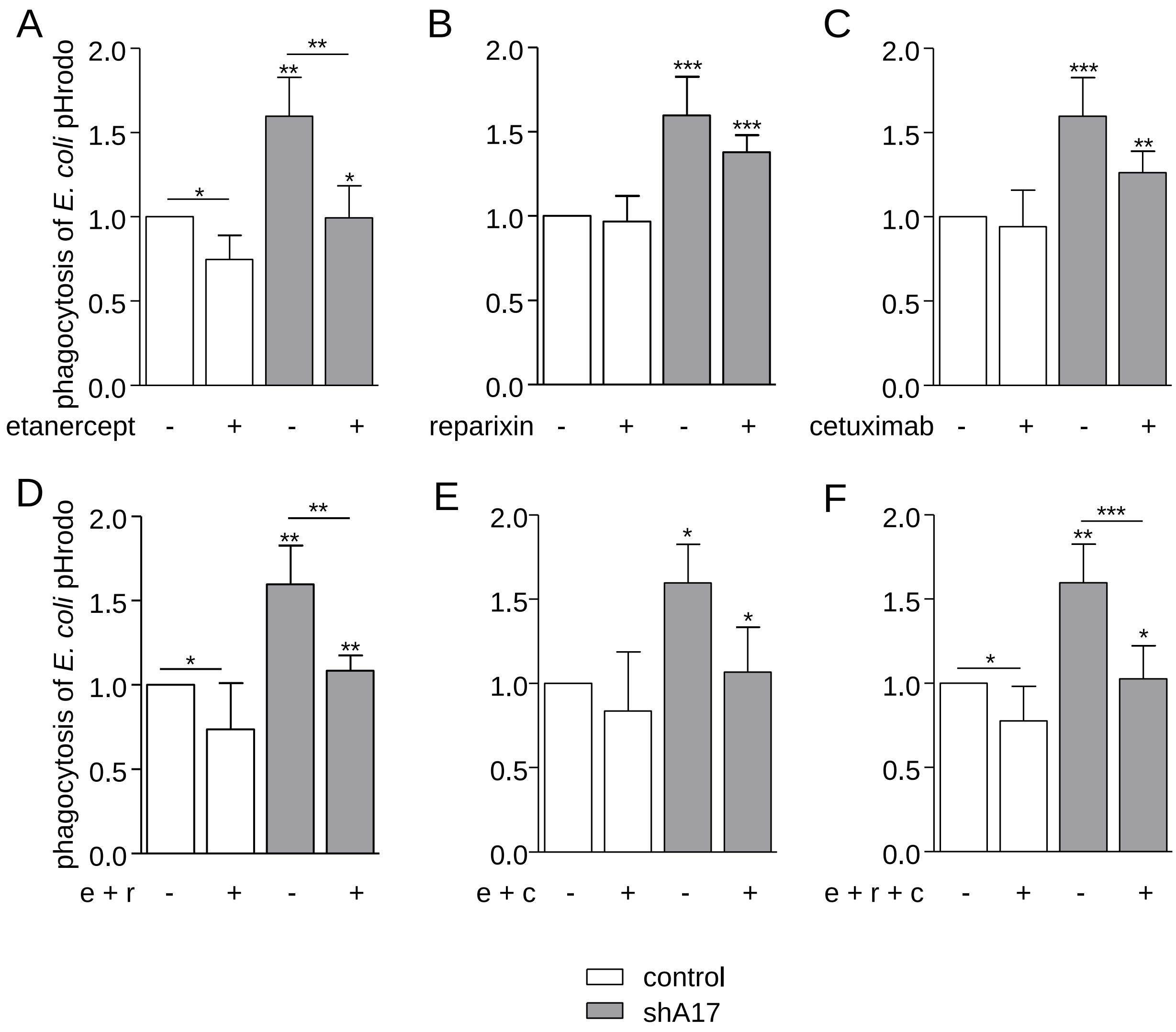

Supplement: Supplementary file 1 [file ijms-21-05978-s001.pdf]
